# Quantum confined two-dimensional electron/hole gas switching by facet orientation of perovskite oxides†

**DOI:** 10.1039/c8ra03928c

**Published:** 2018-06-05

**Authors:** Fei Zhou, Yong Liu, Zhonghong Lai, Mingqing Liao, Yi Zhou, Yudong Huang, Jingchuan Zhu

**Affiliations:** School of Materials Science and Engineering, Harbin Institute of Technology 150001 Harbin China fgms@hit.edu.cn; MIIT Key Laboratory of Critical Materials Technology for New Energy Conversion and Storage, School of Chemistry and Chemical Engineering, Harbin Institute of Technology 150001 Harbin China; National Key Laboratory of Science and Technology on Advanced Composites in Special Environments, Harbin Institute of Technology 150001 Harbin China; National Key Laboratory for Precision Hot Processing of Metals, Harbin Institute of Technology 150001 Harbin China; Analysis and Testing Center, Harbin Institute of Technology 150001 Harbin China

## Abstract

The Polar discontinuity at heterointerface and the bare surface reconstructs the electronic phase of perovskite oxides. This gives rise to confined free electrons which intrinsically transit material from band insulator to metal. However, the insulator–metal transition induced by free holes has not been investigated so far due to the challenge in obtaining free hole state in oxides. Here, we propose a simple method whereby free holes can be obtained *via* polar facet reorientation. In the high polarity case, free holes can be supported by the lift up of O 2p subbands, which split into three independent subbands (one heavy hole subband and two light hole subbands) due to strong quantum confinement. Results show that both of the free electron and hole states are confined in a two dimensional quantum well, subjecting to the confined energy (*E* − *E*_F_) and occupied density of states around the Fermi level, indicating a finite thickness for preserving the metal states.

## Abstract 2

The Polar discontinuity at heterointerface and the bare surface reconstructs the electronic phase of perovskite oxides.

## Graphical abstract

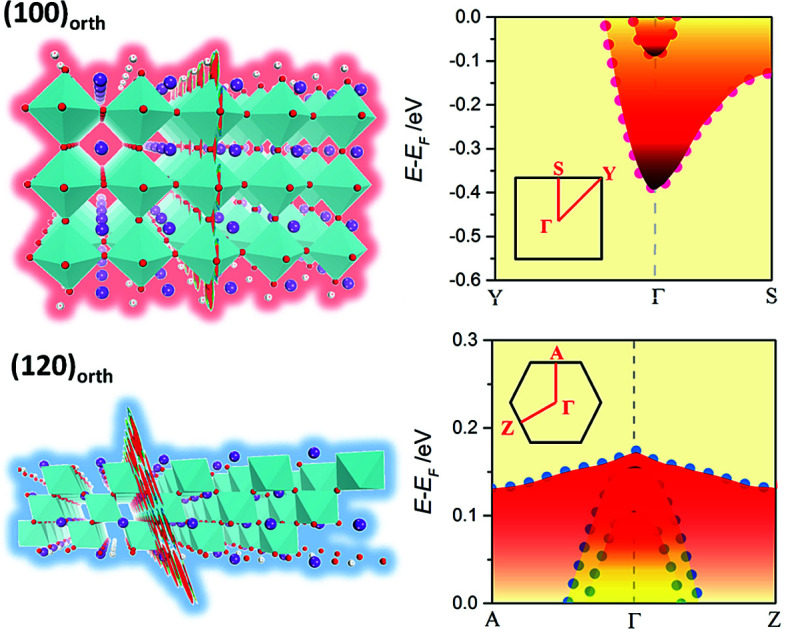

## Introduction

Two-dimensional electron/hole gases (2DEGs/2DHGs) were first studied at the interfaces of semiconductor heterostructures.^1,2^ Recent studies have revealed the existence of high mobility 2DEGs at LaAlO_3_/SrTiO_3_ interface^3^ and even at the bare surface of SrTiO_3_,^4,5^ which are treated as the cornerstone of oxide electronics.^6^ Electronic reconstruction at interfaces or surfaces that occur to compensate for the polar discontinuity of adjacent constituents plays a key role in the formation of localized 2DEGs.^7^ This gives rise to attractive physics, such as insulating-metallic state transitions,^8^ superconductivity,^9^ spiral magnetism and giant negative magnetoresistance.^10–12^ These above-mentioned examples occur on n-type interface/surface, such as the interface construction of (LaO)^+^/(TiO_2_)^0^ or with defects created on bare SrTiO_3_ surface, leading to the mixed dimensionality of confined conducting electrons.^13^ On the contrary, it is still challenging to obtain the free holes at interfaces or surfaces of oxides,^3,14^ although recent experiment shows that strong hole carriers can be seen at the LaAlO_3_/SrTiO_3_ heterointerface by reducing the oxygen defects.^15,16^ However, 2DHGs have been researched in Ge/Si based semiconductor heterostructures, presenting novel physics, such as spin Hall effect induced edge-spin accumulation and well-controlled Coulomb blockade oscillations.^17–19^ The lack of investigations of 2DHGs on perovskite oxides might be ascribed to the limitation of current epitaxial growth technology, which generally allows films to grow along (001)_cubic_ direction through substrate matching, preserving an extra e^−^/2 per unit cell on the discontinued interface or surface. Our recent experiments show that 2D potassium niobates can be grown automatically in liquid with facets of 60° tilting of oxygen octahedrons, providing a practical basis for the study of the facets effects to 2DEGs/2DHGs.

In this letter, *ab initio* calculation is used to investigate the confined 2DEGs/2DHGs in 2D KNbO_3_. Results show that the increase of polar discontinuity by facets engineering would change the electronic phase of the material surface, leading to the formation of switchable 2DEGs and 2DHGs on 2D KNbO_3_ structures by varying facet orientations. These free electron/hole states around the Fermi level (*E*_F_) are strongly quantum localized. This concept not only endows the material with an insulator–metal transition, but also provide an opportunity for manipulating 2DEGs/2DHGs in the quantum confined regime.

### Computational details

The first-principle calculation is performed with the Cambridge Serial Total Energy Package.^20^ 2D crystal models are fully relaxed through geometry optimization according to system energy, force and stress by DFT with the Perdew, Burke, and Ernzerhof functional.^21^ For our main objective is qualitatively investigating the behaviors of quantum confined free electronic states, ultrasoft pseudopotentials are applied to describe the ionic cores with a plane-wave cutoff energy of 340 eV. The *k*-point sampling grids are 3 × 3 × 1 for 2D crystal slabs and 4 × 4 × 2 for bulk KNbO_3_ crystal, respectively. The vacuum spacing layer between neighboring repeat units of 2D slabs are set to 1 nm. The truncated surface are capped by H atoms, in order to neutralize the surface dangling oxygen bonds.

The surface energy *E*_s_ is computed using the formula *E*_s_ = (*E*_slab_ − *nE*_bulk_)/2*A*,^22^ where *E*_slab_ and *E*_bulk_ are the total energy of the 2D slab and the bulk per unit cell, respectively. They are all obtained by DFT calculation. *n* is the number of bulk unit cell contained in the 2D slab. 2*A* is the surface area of each 2D slabs.

## Results and discussion

### 2DEGs/2DHGs switching

**Fig. 1:**
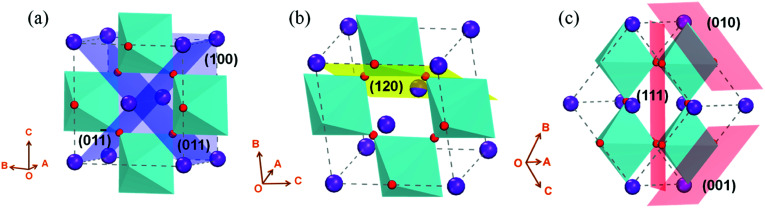
(a) (100), (011) and (011̄) facets of *Amm*2 orthorhombic KNbO_3_ (cut plane marked by blue) with 0° tilt of oxygen octahedrons, these three facets are perpendicular to each other, forming a cubic morphology. (b) (120) facet (cut plane marked by yellow) with 60° tilt of oxygen octahedrons, shows a pseudo-hexagonal symmetry in plane. (c) (001), (010) and (111) facets (cut plane marked by red) with 90° tilt of oxygen octahedrons.

KNbO_3_ is a typical orthorhombic ferroelectric with K^+^ and Nb^5+^, showing different valence discontinuity from conventional SrTiO_3_ in various facets orientation. In order to know the facets differences of orthorhombic KNbO_3_, density functional theory is used. Two dimensional (2D) slabs with tilted oxygen octahedrons in angles of 0°, 60°, 90° are built (as sketched in Fig. 1, the 2D slabs used for DFT calculation see Fig. S1†). Calculated results show that the facets of (100)_orth_, (011)_orth_ and (011̄)_orth_ (0° tilting of oxygen octahedron, see Fig. 1a), which are perpendicular to one another with a terminal plane of (KO)^−^, have small surface energy (*E*_s_) below 100 MeV Å^−2^ (see Table 1). Lower surface energies explains why KNbO_3_ crystals always grow as cubic particle in hydrostatic reactions. These facets correspond to the (100)_cub_, (010)_cub_ and (001)_cub_ oriented facets in cubic SrTiO_3_ respectively, but with distinct valence difference in alternate atomic layers, (SrO)^0^/(TiO_2_)^0^ in SrTiO_3_ and (KO)^−^/(NbO_2_)^+^ in KNbO_3_. Besides 0° tilted octahedron 2D models, the *E*_s_ of 60° and 90° tilted models (Fig. 1b and c) are all between 150 MeV Å^−2^ and 300 MeV Å^−2^, maintaining a strong energy barrier in self-driven growth processes. The large differences in surface energies will work on the formation of stable surface structures of 2D KNbO_3_. This further results in their distinct electronic reconstructions on the surface.

**Table 1:** The energy difference of 2D slab to bulk (*E*_slab_ − *E*_bulk_), surface areas (*S*), surface energy (*E*_s_) of 2D KNbO_3_ with different facets

	(100)	(011)	(011̄)	(001)	(010)	(111)	(120)
*E*_slab_ − *E*_bulk_ (eV)	11.68	4.68	3.34	55.68	40.68	44.68	51.68
Surface (Å^2^)	67.446	65.404	65.404	92.224	92.768	140.926	113.338
*E*_s_ (eV Å^−2^)	0.086	0.033	0.026	0.298	0.217	0.158	0.227

**Fig. 2:**
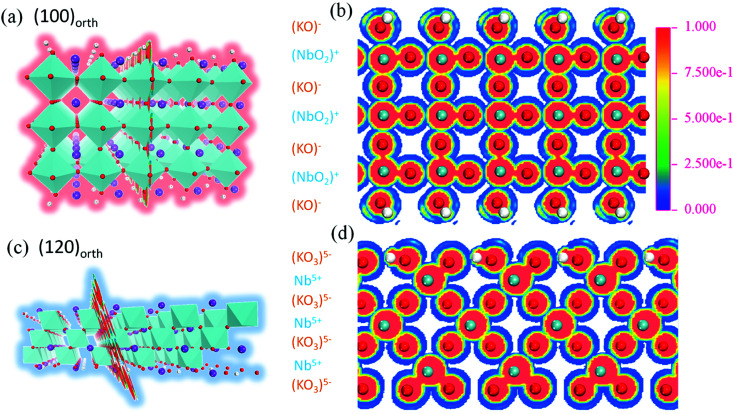
(a) and (c) The trilayers (octahedron layer) atomic structures of (100)_orth_ and (120)_orth_ oriented 2D KNbO_3_ models, surface dangling bonds of oxygen were capped by hydrogen atoms, the selected cutting slices of Nb–O plane of calculated ELF in (a) and (c) were showed in (b) and (d) by colored maps, respectively. Both of the 2D structures were obtained by energy minimization through optimizing geometry.

**Fig. 3:**
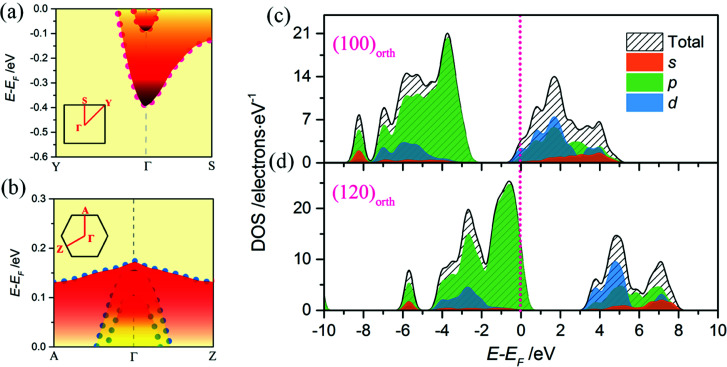
(a) and (b) Calculated band structures of trilayers (octahedron layer) (100)_orth_ and (120)_orth_ oriented 2D KNbO_3_ models, respectively. Free electron states switch to free holes when facets changed. (c) and (d) Partial density of states of s, p, d orbits of both of 2D KNbO_3_ models, respectively. Oblique fill lines show the sum of DOS of different orbits.

To investigate the relevance of surface energy to surface polar properties, trilayers 2D models of KNbO_3_ crystal with (100)_orth_ and (120)_orth_ facet orientations are built. After lattice relaxation, stable 2D structures are obtained (Fig. 2a and c). Their electronic structures are calculated. Specifically, the 60° tilted facet of (120)_orth_ 2D crystal shows an alternating valence variation of (KO_3_)^5−^/Nb^5+^, indicating a potential huge polar difference in alternating atomic planes, as shown in Fig. 2d. From the sliced mapping of electron localization functions (ELF) in Fig. 2b and d, quantum confined surface states can be clearly observed on both surfaces, which surround the perfectly localized core and the bonding electrons (where ELF approaches 1)^23^ of oxygen atoms, allowing the electron and hole carriers moving freely on the surface. In consideration of the distinct electronic reconstructions, (100)_orth_ with extra e^−^/2 (N-type) and (120)_orth_ with extra 3h^+^/2 (P-type) injection per unit cell, their electronic phases should be different. Fig. 3a and b show the calculated bands around *E*_F_ of (100)_orth_ and (120)_orth_ oriented 2D KNbO_3_. Conventional free electron conductance switches to free hole conductance when a facet of (100)_orth_ changes to (120)_orth_ by the 60° tilting of oxygen octahedrons. The preferential growth facet also changes the symmetry of electronic states transitions, resulting in an indirect optical band gap transition changing to direct at *Γ* point, as the red arrows indicated in ESI Fig. S2a and S2b.† Density of states of s, p, d orbits (Fig. 3c and d) shows further insights of this switching behavior. The potential of low-lying conduction band descends by 0.39 eV due to extra electron injection, pushing the Nb 4d band to *E*_F_ and allowing electrons to move freely at the bottom of the conduction band (Fig. 3c). Conversely, extra holes injection induced by strongly quantized O 2p states lifts the valence band by 0.17 eV above *E*_F_, giving free holes. The low-lying subbands of Nb 4d are split t_2g_ bands, composed of a heavy (d_*yz*_ doublet) and a light (d_*xy*_ singlet) band.^24^ Strong confinement lifts up the light t_2g_ band with respect to the heavy ones (Fig. 3a), demonstrated in the thickness dependence of band structures variations of (100)_orth_ models (See Fig. S4†), due to the difference in *m*_e_. The top O 2p bands also split into three subbands, the highest heavy (*E*_hb_^+^) and underlying two light (*E*_lb_^+^) ones (Fig. 3b) under the quantum confined regime.

### Quantized electronic states in a 2D square quantum well

**Fig. 4:**
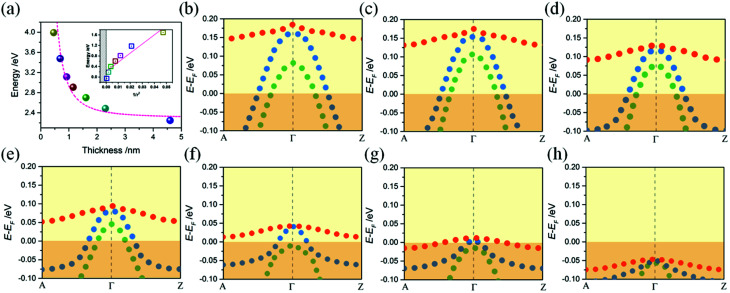
(a) Colored balls correspond to the calculated band gap energy of 2D models with thicknesses varying from 0.46 nm to 4.6 nm. The abrupt increase in band gap energy occurs at below 2 nanometer size, where there is a presence of strong quantum confinement. The theoretically calculated curve (pink dotted line) fits very well to the experimental data. Inset illustrates the linear relationship between the quantum confinement effected energy values and 1/*r*_2_. (b)–(h) The evolution of quantum localized hole states with 2D well thickness varying. Red dotted line denotes the heavy hole (*E*_hb_^+^) band, blue and green dotted lines denote the two light (*E*_lb1_^+^ and *E*_lb2_^+^) bands.

To obtain the effect of the size limitation to free electronic states, the band structures of (120)_orth_ 2D KNbO_3_ with thicknesses that range from 0.23 nm (1 octahedron layer) to 4.6 nm (20 octahedron layers) are calculated (see Fig. S8†). Strong quantum confinement can be observed with a decrease in thickness, accompanied by the splitting of degenerate energy into independent sub-bands, as indicated by the red arrow in ESI Fig. S8.† A two-dimensional square quantum well is used to describe the thickness dependence of quantum confined band gaps, given by: 
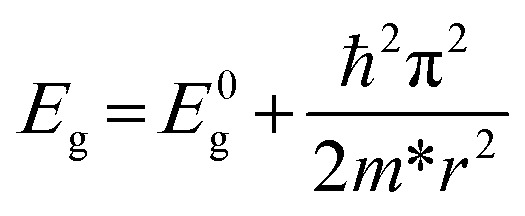
,^25^ where *E*_g_ is the calculated energy gap of the 2D model and *E*^0^_g_ is the calculated energy gap of the bulk crystal model (*E*^0^_g_ ∼ 2.3 eV, as shown in ESI Fig. S9†). The second term represents quantum localization, where *ℏ* is the reduced Planck's constant, *r* is the sheet thickness, and 
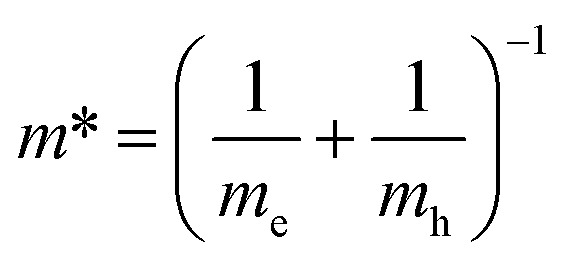
 is the reduced effective mass. Fig. 4a plots the calculated thickness dependence of quantum well transition energies with dimension reduction. The value of m* is 0.6 *m*_e_, which is obtained from fitting a best fit line to calculate *E*_g_ values (pink dotted line in Fig. 4a). The solid fit line in the inset confirms the linear relationship of quantum confined energies to 1/*r*^2^ for a 2D square quantum well.

The hole states occupation of samples are shown in Fig. 4b–h. A monotonous decreasing relationship of confined energy of hole states with respect to the thicknesses of 2D wells is observed, where the O 2p bands will be totally under *E*_F_ when thickness approaches 4.6 nm. Strong confinement leads to the splitting of the top O 2p band into one heavy (*E*_hb_^+^, denoted as red dots) and two light (*E*_lb1_^+^ and *E*_lb2_^+^, denoted as blue and green dots respectively) bands at *Γ* point. The decrease in quantum confined energy contributes to the appearance of degenerate *E*_hb_^+^ and *E*_lb1_^+^ bands (as shown in Fig. 4b–f), accompanied by the gradual increase of *m*_h_ of *E*_lb1_^+^ along momentum *k*. Until reaching the weak confined situation (Fig. 4g), the light *E*_lb2_^+^ band descends down to *E*_F_ first, then degenerate *E*_hb_^+^ and *E*_lb1_^+^ bands will further combine with *E*_lb2_^+^ and totally descend under *E*_F_ (Fig. 4h). A similar trend of low-lying conduction t_2g_ band of (100)_orth_ models being lifted up to *E*_F_ can be observed with an increase in thickness (see ESI Fig. S5†), showing that both of 2DEG and 2DHG cases have strong quantum confinement along the *Z* direction.

### Insulator–metal transition

**Fig. 5:**
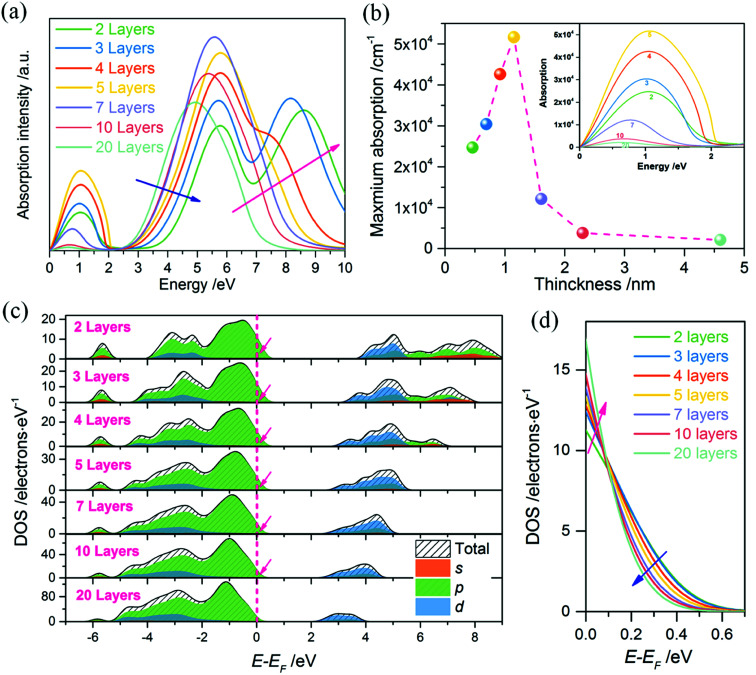
(a) Absorption properties of (120)_orth_ oriented 2D KNbO_3_ models, the absorption in low frequency indicates an insulator–metal transition absorption. (b) The thickness dependences of absolute absorption intensity of free hole states, inset is the zoom in of low-frequency range. (c) PDOS of (120)_orth_ oriented 2D KNbO_3_ with thickness range from 2 layers to 20 layers, show the thickness dependence of quantum localized free holes and the corresponding contributions of different orbits. (d) Evolution trend of quantized hole states with respect to the thickness varying. The quantum localized energy and the absolute value of electronic states per electron volt co-work on the free hole states absorption, showing a converse contribution (as the blue and pink arrows indicating with reverse directions).

To obtain further insight of the insulator–metal transition of our 2DFEG/2DFHG systems, optical absorption properties of models with varying thicknesses are calculated. Fig. 5a shows the absorption spectra of (120)_orth_ oriented 2D KNbO_3_ models (thicknesses are from 0.46 nm to 4.6 nm). The strong absorption across the whole optical gap demonstrates the insulator–metal transition. Fig. 5b plots the maximum absorption of the free hole states (collected from low-frequency absorption spectra, as shown in inset figure), showing that the free holes absorption intensity increase gradually with an increase in thickness. Up to above 1.15 nm (5 layers), it decreased dramatically with an increase in thickness, indicating a critical thickness of about 2 nm for the existence of metallic states for 2DHGs. A gradual blue-shift of intrinsic absorption band edges can be observed when the number of layers increases (indicated by the blue arrow in Fig. 5a), along with one single peak extending to two individual peaks (as pink arrow indicated in Fig. 5a) due to the splitting and lifting of s, p orbits from the degenerate d band (Fig. 5c). This agrees with the quantized characters of conduction band in 2D quantum well. Although the occupation energy of hole states above *E*_F_ decreases with an increase in well thickness (such as the pink arrow marked in Fig. 5c and blue arrow marked in Fig. 5d), the increase of DOS near *E*_F_ (marked by pink arrow in Fig. 5d) competes with the decrease of confined energy, leading to an increase initially then decrease of free hole states absorptions. The increase in DOS near *E*_F_ is ascribed to the increased total numbers of atoms per unit area when stacking layers increase. However, the confined energy still plays a dominant role for metallic states maintaining, as plotted in Fig. 4b. The absorption properties of free electrons ((100)_orth_ oriented 2D KNbO_3_ models) show a similar trend of confined states transition while the thickness variation (see ESI Fig. S6a†), although the ∼4 nm (10 layers) model shows an anomalous absorption in the low-frequency range, which can be ascribe to the abrupt increase of the local DOS near *E*_F_, as marked by pink arrow in ESI Fig. S6b.†

## Conclusions

In summary, beyond well-known concepts for rearranging these surface electronic states (with extra electrons injecting) in 2D heterointerface or bare surface, our results show that additional holes injection can be maintained by the increase of surface polarity. The 2DEGs/2DHGs switching can be simply fulfilled by facet orientations. Distinct orientations change the polar discontinuity of alternating atom layers, corresponding to that huge surface energy differences. The thickness dependent behaviors of free electron/hole states subjecting to quantum confinement are addressed by the varied thickness of 2D models. Compared with free electron states induced insulator–metal transition (4 nm sample still exists strong free electron states absorption), free hole states transition depends much stronger on the thickness of 2D quantum well, with a small critical value of ∼2 nm. The formation of free electrons is more complex than free holes (contributed by O 2p states) due to the hybridization of s, p, d orbits in conduction bands. Such as one p band abnormally extends under *E*_F_ (among the split t_2g_ bands) for (100)_orth_ oriented monolayer model at the extreme confined regime, acting to form an additional free electron conduction band (see ESI Fig. S5a†). Despite their differences, both of 2DEGs and 2DHGs are resulted from strong quantum confinement. Furthermore, the confined energy (*E* − *E*_F_) and the total DOS occupying near *E*_F_ (which varies with sample thickness) jointly act on the metallic state transition, demonstrating that metallic states can be modulated not only by the amount of donor-like defects or adsorbates, but also simply by thickness variation.

In contrast to previous theoretical and experimental results,^3,4,24,26^ our theoretical study reveals that the free holes can be obtained on high polar surface of perovskite oxides, gives the origins of oxides 2DEGs/2DHGs, including their intrinsic switching by surface polarity change and confined evolution behaviors with respect to quantum well thickness, emerging as a promising basis towards design the new generation all-oxides electronic devices.

## Conflicts of interest

There are no conflicts to declare.

## Supplementary Material

RA-008-C8RA03928C-s001
