# Supplementary material for: Quantum confined two-dimensional electron/hole gas switching by facet orientation of perovskite oxides
Source: RSC Adv. 2018 Jun 5;8(37):20477–82. doi: 10.1039/c8ra03928c (PMC9080832; doi:10.1039/c8ra03928c)
Supplement: RA-008-C8RA03928C-s001 [file RA-008-C8RA03928C-s001.pdf]

Electronic Supplementary Material (ESI) for RSC Advances.
This journal is © The Royal Society of Chemistry 2018

Supporting Information

Quantum confined two-dimensional electron/hole gas switching by facet orientation of perovskite oxides

Fei Zhou,[†,‡] Yong Liu,[†,§] Zhonghong Lai,[⊥] Mingqing Liao,[†] Yi Zhou,[†] Yudong Huang,[‡] and Jingchuan Zhu[*,†,§,‖]

[†] School of Materials Science and Engineering, Harbin Institute of Technology, 150001, Harbin, China
[‡] MIIT Key Laboratory of Critical Materials Technology for New Energy Conversion and Storage, School of Chemistry and Chemical Engineering, Harbin Institute of Technology, 150001, Harbin, China
[§] National Key Laboratory of Science and Technology on Advanced Composites in Special Environments, Harbin Institute of Technology, 150001, Harbin, China
[‖] National Key Laboratory for Precision Hot Processing of Metals, Harbin Institute of Technology, 150001, Harbin, China
[⊥] Analysis and Testing Center, Harbin Institute of Technology, 150001, Harbin, China

[*] E-mail: fgms@hit.edu.cn

1. Computational methods

The first-principle calculation is performed with the Cambridge Serial Total Energy Package[1]. 2D crystal models are fully relaxed through geometry optimization according to system energy, force and stress by DFT with the Perdew, Burke, and Ernzerhof functional[2]. For our main objective is qualitatively investigating the behaviors of quantum confined free electronic states, ultrasoft pseudopotentials are applied to describe the ionic cores with a plane-wave cutoff energy of 340 eV. The *k*-point sampling grids are 3×3×1 for 2D crystal slabs and 4×4×2 for bulk $KNbO_3$ crystal, respectively. The vacuum spacing layer between neighboring repeat units of 2D slabs are set to 1 nm. The truncated surface are capped by H atoms, in order to neutralize the surface dangling oxygen bonds.

The surface energy E_s is computed using the formula $E_s = (E_{slab} - nE_{bulk}) / 2A$ [3], where E_{slab} and E_{bulk} are the total energy of the 2D slab and the bulk per unit cell, respectively. They are all obtained by DFT calculation. n is the number of bulk unit cell contained in the 2D slab. $2A$ is the surface area of each 2D slabs.

2. Surface crystal models of different facets

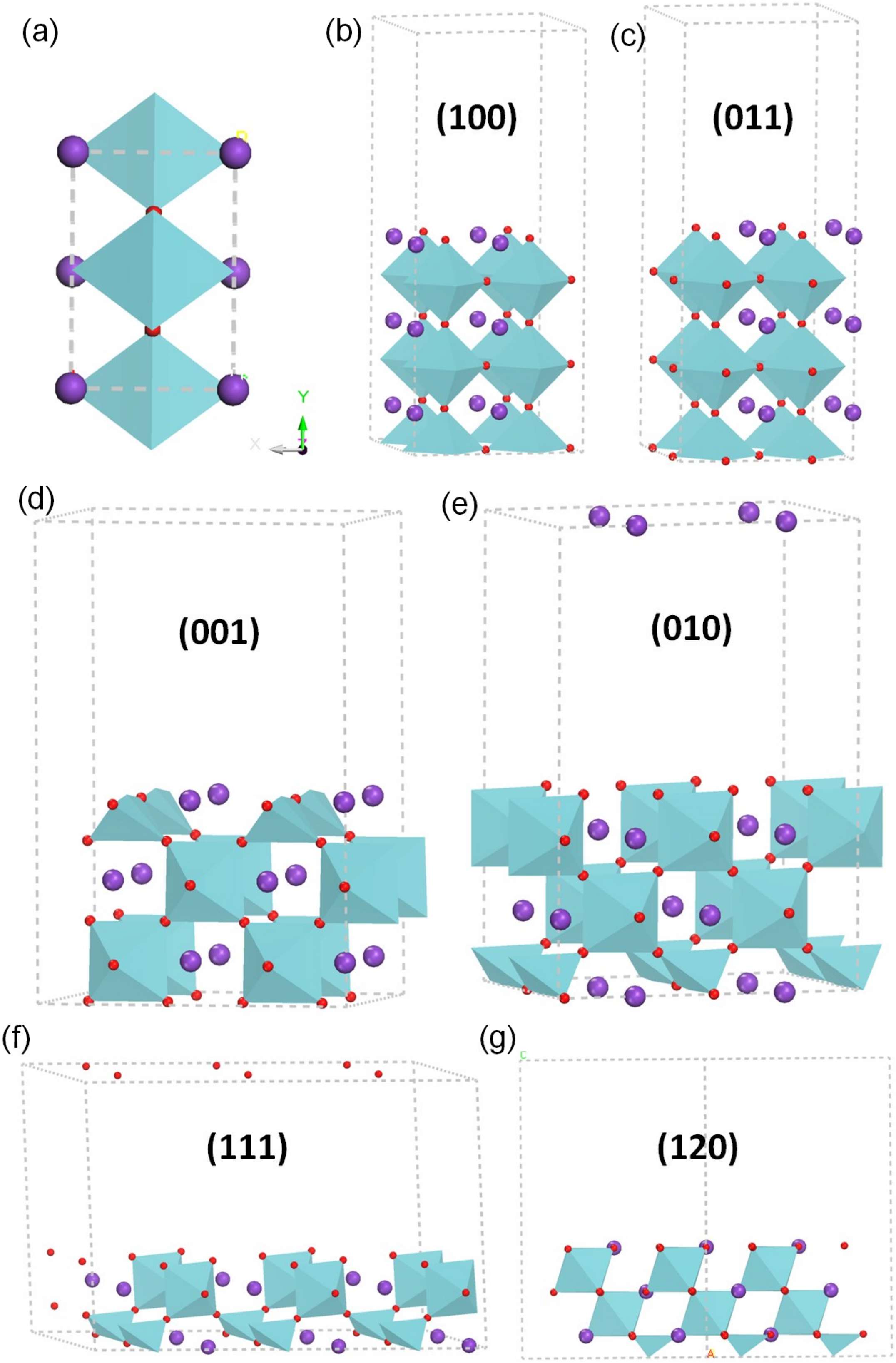

Fig. S1 (a) Bulk crystal model of Amm2 orthorhombic $KNbO_3$; (b)-(g) Two-dimensional crystal models of surface of (100), (011), (001), (010), (111), (120) facets.

3. Band structure of trilayers models of 2D $KNbO_3$

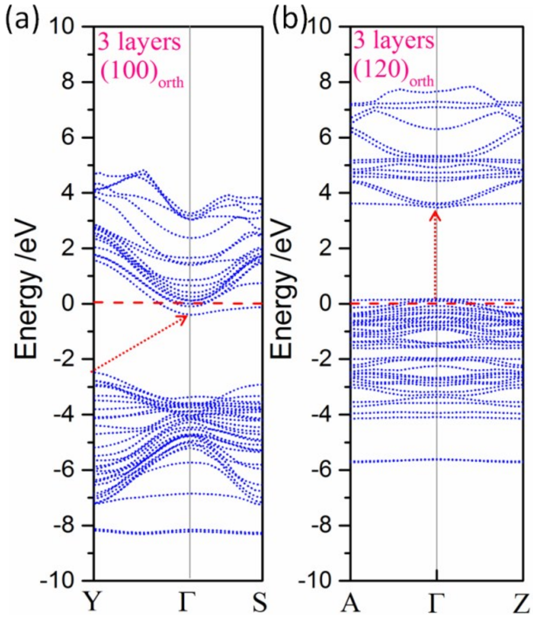

Fig. S2 (a) Band structure of (100) facet 2D $KNbO_3$, the conductive t_{2g} band descend below E_F allowing the electrons moving freely on these bands. The minimum optical band gap shows that the electronic states transit from Y to Γ (indicated by red arrows), indicating a indirect band transition. (b) Band structure of (120) facet 2D $KNbO_3$, the O 2p subbands lift up above E_F showing as free hole conduction. The preferential geometric structure changes the symmetry of electronic state transition, where (120) oriented facet showing a direct band transition, as indicated by red arrow.

4. Geometric structure models of $(100)_{orth}$ oriented 2D crystal

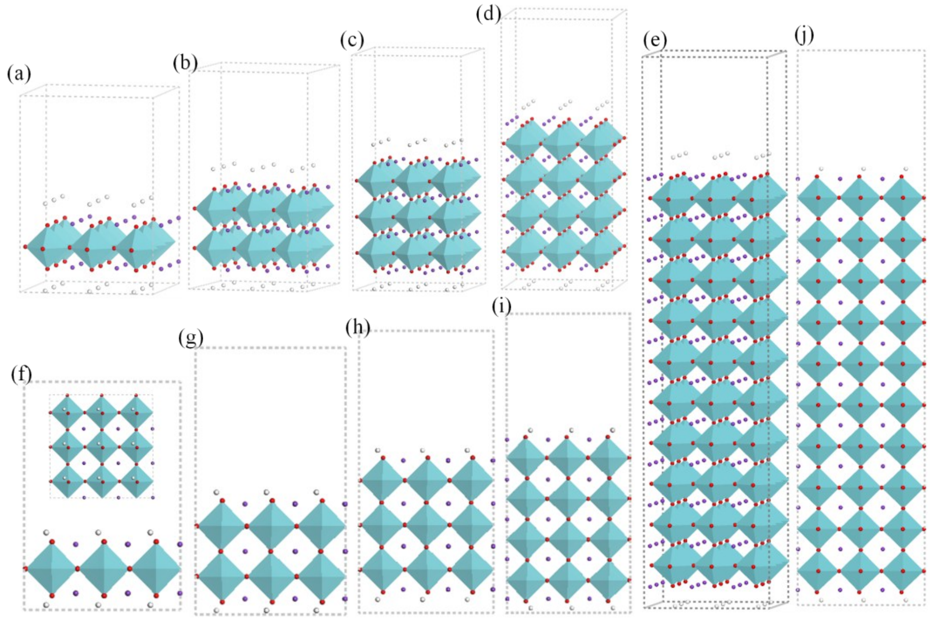

Fig. S3 (a)-(e) As built models of 2D crystal with $(100)_{orth}$ orientation, their thickness varies from monolayer (0.4 nm) to ten layers (4 nm); (f)-(j) Stable crystal structure of (a)-(e) after fully lattice relaxation by DFT.

5. Calculated band structures of $(100)_{orth}$ oriented 2D crystal

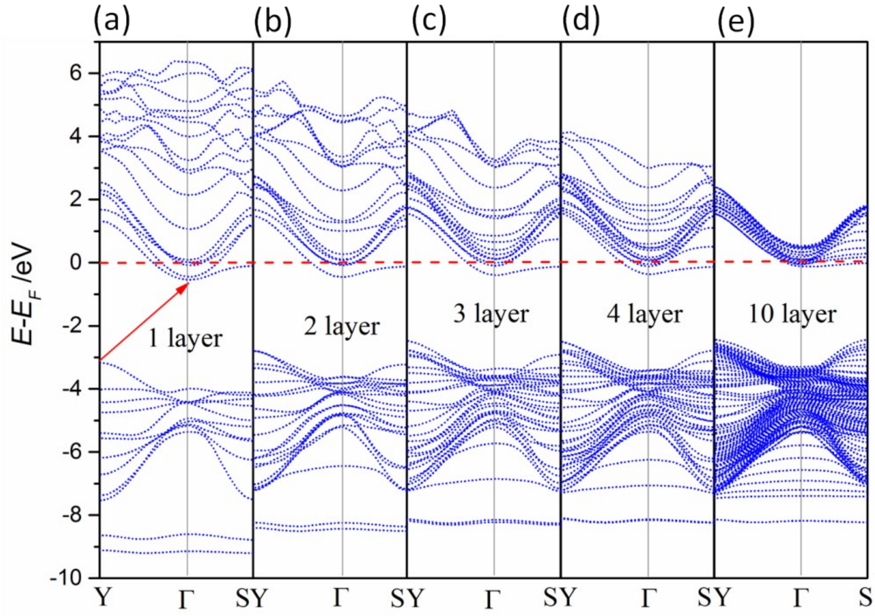

Fig. S4 (a)-(e) Band structures of (100) facet 2D $KNbO_3$ with thickness range from monolayer (0.4 nm) to ten layers (4 nm). All of them show indirect band transition. Their t_{2g} band descend below E_F allowing the electrons moving freely on these bands. The optical band gap and the confined energy (E-E_F) decrease gradually with an increase in thicknesses. The increase of quantum confinement leads to degenerate conduction band splitting into independent sub-bands.

6. Free electronic states occupation below E_F of $(100)_{orth}$ oriented 2D crystals

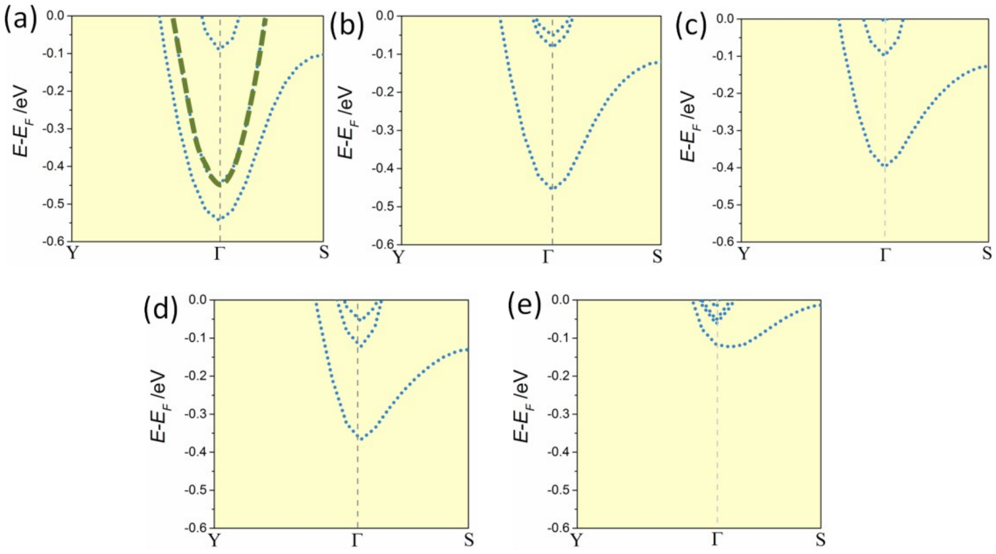

Fig. S5. (a)-(e) Free electronic states occupation of (100) facet 2D $KNbO_3$ with thickness range from monolayer (0.4 nm) to ten layers (4 nm). The confined energy (E-E_F) decrease gradually with an increase in thickness. Under strong quantum confinement, One of the p band will extend to low energy (between split t_{2g} band), presenting as additional free electrons conductive band (marked by green dash line in figure S5(a)).

7. Insulator-metal transition behavior of free electrons absorptions of $(100)_{orth}$ oriented 2D $KNbO_3$.

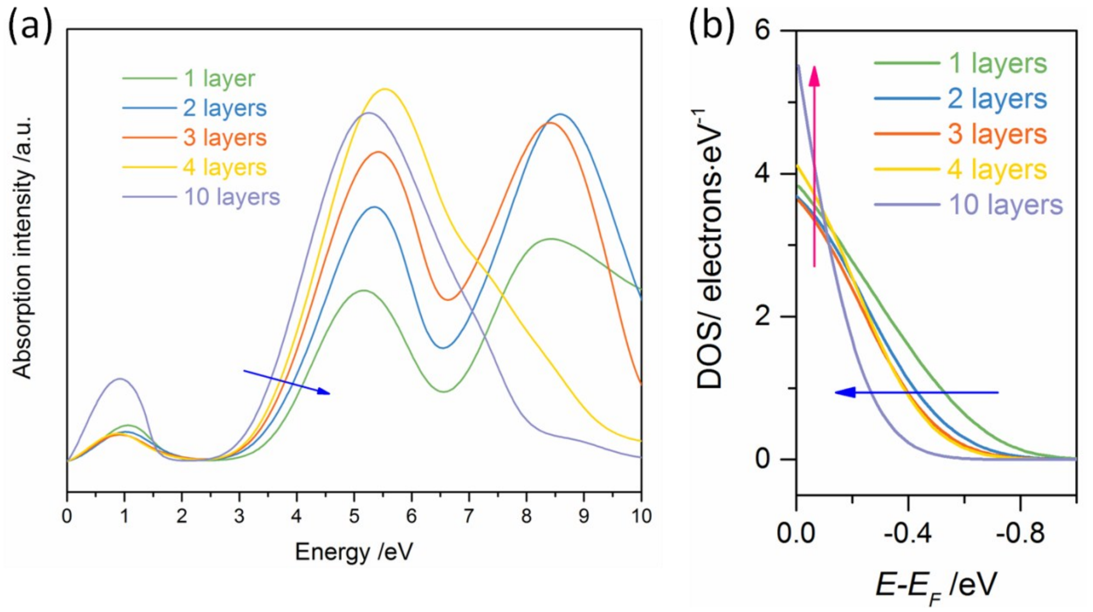

Fig. S6. (a) Absorption properties of $(100)_{orth}$ oriented 2D $KNbO_3$ models, the absorption in low frequency indicates an insulator-metal transition absorption. Strong quantum confinement can be observed from the variation of absorption band edges of models with a decrease in thickness. The anomalous absorption of ten layers model in the low-frequency range can be ascribe to the abrupt increase of the local DOS near E_F, as the red arrow indicated in (b).

8. Geometric structure models of $(120)_{orth}$ oriented 2D crystal

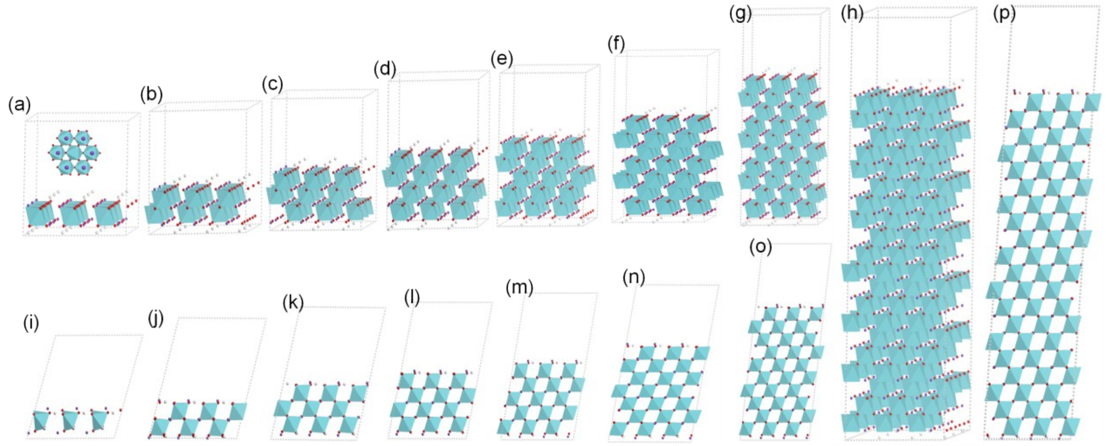

Figure S7. (a)-(h) As built models of 2D crystal with $(120)_{orth}$ orientation, their thickness varies from monolayer (0.23 nm) to twenty layers (4.6 nm); (i)-(p) Stable crystal structure of (a)-(h) after fully lattice relaxation by DFT. A strong lattice distortion occurs after total energy minimization of monolayer model (comparing the (a) and (i)), showing a structural instability of mono-octahedral layer model with $(120)_{orth}$ orientation.

9. Calculated band structures of $(120)_{orth}$ oriented 2D crystal

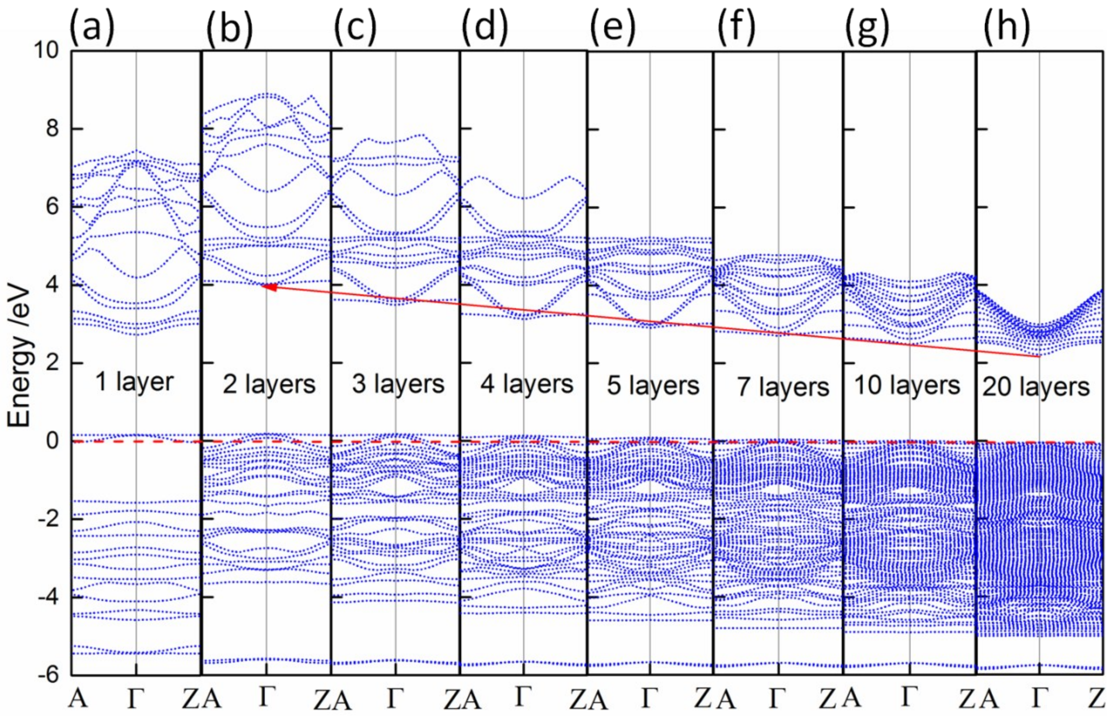

Fig. S8 (a)-(h) Band structures of (120) facet 2D $KNbO_3$ with thickness range from monolayer (0.23 nm) to twenty layers (4.6 nm). All of them show a direct optical band transition. Their O 2*p* band lift up above E_F allowing the holes moving freely on these bands. The optical band gap and the confined energy (E-E_F) decrease gradually with an increase in thicknesses. The abnormal band structure of the mono-octahedral layer (a) can be ascribed to the anomaly of its geometric structure in figure S7(i).

10. Calculated electronic structures of bulk $KNbO_3$ crystal

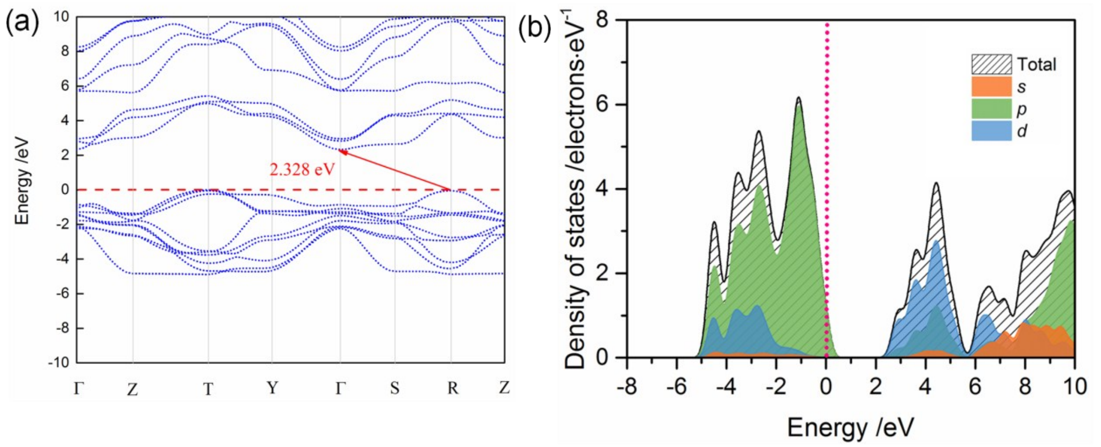

Fig. S9. (a) Calculated band structure of Amm2 orthorhombic $KNbO_3$ crystal. An indirect band gap of 2.328 eV can be obtained by the PBE functional calculation. (b) Calculated PDOS of $KNbO_3$ crystal, showing that the conduction band is constructed by the hybridization of *p*, *d* states, the valence band is constructed by O 2p states.

References

1. Segall, M. D.; Lindan, P. J. D.; Probert, M. J.; Pickard, C. J.; Hasnip, P. J.; Clark, S. J.; Payne, M. C. *J. Phys.: Condens. Matter.* **2002,** 14, (11), 2717.
2. Perdew, J. P.; Burke, K.; Ernzerhof, M. *Phys.Rev.Lett.* **1996,** 77, (18), 3865-3868.
3. Bi, Y.; Ouyang, S.; Umezawa, N.; Cao, J.; Ye, J. *J.Am.Chem.Soc.* **2011,** 133, (17), 6490-2.
